# Characterization and haplotype study of 6 novel STR markers related to the KCNQ1 gene in heterogeneous cardiovascular disorders in the Iranian population

**DOI:** 10.3906/sag-1805-43

**Published:** 2019-04-18

**Authors:** Azam AMIRIAN, Zahra ZAFARI, Zohreh SHARIFI, Alireza KORDAFSHARI, Morteza KARIMIPOOR, Sirous ZEINALI

**Affiliations:** 1 Department of Molecular Medicine, Biotechnology Research Center, Pasteur Institute of Iran, Tehran Iran; 2 Department of Biology, Shahed University, Tehran Iran; 3 Medical Genetics Laboratory, Kawsar Human Genetics Research Center, Tehran Iran

**Keywords:** Allele heterozygosity, cardiac disease, haplotype, Iran, STR marker

## Abstract

The *KCNQ1* gene has a significant role in long QT syndrome, Jervell and Lange-Nielsen syndrome, familial atrial fibrillation, and short QT syndrome. Analyzing such heterogeneous disorders, six novel short tandem repeat (STR) markers around the *KCNQ1* gene were found and evaluated in a healthy population, and other statistical traits of the markers were detected.**Materials and methods:** Using Tandem Repeats Finder (TRF) and Sequence-Based Estimation of Repeat Variability (SERV) software, STR markers were detected with valid tetra- and pentanucleotide repeats. The markers were investigated for a total of 60 unrelated Iranian healthy individuals and analyzed using GenAlEx 6.502 and Cervus 3.0.7.**Results:** A total of 77 haplotypes was detected, of which 25 haplotypes were unique and the others occurred at least two times. The number of haplotypes per locus ranged from 7 to 18 with the highest frequency of 69.2%, and the observed heterozygosity was calculated as 0.589. The power of discrimination ranged from 0.70 to 0.96. Five of the markers meet Hardy–Weinberg equilibrium.**Conclusion: **A novel panel of STR markers was described with high allele heterozygosity and segregation in every locus, which may lead to faster and more credible recognition of the disease-inducing *KCNQ1* gene and allow it to be used for human identity testing and prenatal diagnosis.

## 1. Introduction

The *KCNQ1* gene is related to a huge family of genes that encode heart potassium channel protein, the most diverse group of ion channels, that is responsible for the repolarization phase of the cardiac action potential in the voltage dependence of activation (1). The gene product is assumed to be capable of forming a heteromultimer with the other potassium channel protein, mink (encoded by *KCNE1*). Loss of function mutations in the *KCNQ1 *gene,**which induce type 1 long QT syndrome (LQTS1, MIM#192500) (2), the most common type of LQTS, cause delayed rectifier potassium current (IKs) in the cardiomyocytes (3) and inner ear (4).

In addition to LQTS, mutations in this gene are also associated with other forms of inherited arrhythmias such as Jervell and Lange-Nielsen syndrome (JLNS), familial atrial fibrillation (AF), and short QT syndrome (SQTS) (5). The gene is located in a region of chromosome 11 that contains a large number of contiguous genes, consisting of 16 coding exons spanning approximately 400 kb (6). Hundreds of different mutations with variable effects on Kv7.1 function have been reported (7). This reveals the considerable clinical importance in LQTS, so linkage studies are the best approach to detection of the mutation for such genetic heterogeneous diseases.

Short tandem repeats (STRs) are highly polymorphic markers and are found in most genomes used in linkage studies (8–11). STRs could also be used for forensic applications, phylogenetic reconstruction, preimplantation genetic diagnosis, and prenatal genetic diagnosis (6,12).

This study set out to explore the utility of STRs in mutated gene diagnosis for LQTs by presenting the six novel tetra- or pentanucleotide STR markers surrounding the *KCNQ1 *gene. Heterozygosity and frequency evaluation of these markers has been carried out in the Iranian population.

## 2. Materials and methods

### 2.1. DNA extraction

Sixty unrelated healthy individuals were selected from the Iranian population. After obtaining informed consent, blood samples were collected in tubes containing ethylenediaminetetraacetic acid (EDTA). The research was approved by the Pasteur Institute of Iran and the local ethics committee. DNA was extracted from blood samples using the KBC Blood-DNA Extraction Kit.

### 2.2. STR marker preparation

The University of California Santa Cruz (UCSC) genome browser, Tandem Repeats Finder (TRF) (13), and SERV (Sequence-Based Estimation of Repeat Variability) sequence programs (14) were used for finding the STR markers. A pentanucleotide, D11SD8.3, and four tetranucleotide (D11SU10.9, D11SU2.2, D11SU0.6, D11SD13.6) tandem repeat markers flanking the *KCNQ1 *gene, plus one tetranucleotide, D11SI, located inside the gene, were selected. 

### 2.3. Marker primer design and amplification 

Primers were designed using Gene Runner software. We used the multiplex PCR method, which amplifies multiple DNA fragments in one polymerase chain reaction (PCR). Each forward primer was labeled with fluorescent-dye labels (either FAM, VIC, or NED dye). STR markers, primer sequences, and their fluorescent-dye labels are shown in Table 1.

**Table 1:** Characteristics of 6 STR markers developed for KCNQ1 gene.

STR loci	Primer sequences (5′–3′)	Dye label	Repeat motifs	Product sizes
D11SU10.9	F: CACCCGTCTGTCTGTCGATTC R: TGTGGAGAAGTTAGTAAGTGGTGAGTG	VIC	(TCCA)10	324–341
D11SU2.2*	F: TAGATAGATGCACAGACAGATCAAAGG R: GGTCTTTCTTCTCAGTTTCCATCC	NED	(ATGG)11	303–346
D11SU0.6	F: TCTCCAGCTTGACCAGACAG R: GTAAAGATACAGGTAGCTAGCTCGGTAG	FAM	(TCCA)12	165–189
D11SI	F: GTACAGAAAGTGCTCCCATCCAC R: GAGGCTTGGTGAGATCGATC	VIC	(TGGA)13	82–123
D11SD8.3**	F: AACCACCACACCAGGATAGTTC R: ATTTGTCTCTGGGTCTCACTTGAC	NED	(TCATC)10	279–320
D11SD13.6	F: ATCCTTTGATGATTAGTGTTTGAGG R: ACAGACTGACTGAATGGAAGATGG	VIC	(TCCA)11	206–247

*The number shows the distance from the gene as 2.2 = 220 kb from the 5ʹ of the gene or D8.3 being 830 kb from the 3ʹ end of the gene. **STRs downstream from the genes were given a name beginning with D, those upstream were given a name beginning with U, and those that were intragenic were given a name beginning with I.

STR loci were reproduced in a single reaction in a volume of 18 µL according to the multiplex PCR method. PCR mixtures and the thermal cycler program are described in Table 2. Fragment amplification was carried out on an ABI 3130XL Genetic Analyzer (Applied Biosystems-Kawsar Biotech Co., Iran). PCR products were examined in Gene-Mapper ID ver. 4.0 (Applied Biosystems).

**Table 2:** Multiplex amplification conditions (cycling and optimized PCR conditions).

Optimized PCR conditions		Cycling and temperature condition	
MgCl2 (50 mM)	3.94 mM	95 °C	5 min
10X PCR buffer	2.8 µL	95 °C	1 min
dNTP (10 mM)	3.2 mM	63 °C	90 s
Distilled water	8 µL	70 °C	1 min
Forward & reverse primer mix	1 µL (1 µL of each 6 pair primers)	35 cycle	
Bovine serum albumin	1 µL	70 ºC	17 min
SmarTaq DNA polymerase	1 µL	Hold	4 °C*
Genomic DNA	1 µL (200 ng/µL)		

* Temperature range (°C).

### 2.4. Statistical significance

Using GenAlEx 6.502 (15), heterozygosity, allelic frequencies, the probability of identity (PI), and the power of exclusion (PE) were estimated. Calculating of the power of discrimination (PD) per locus was performed as a proximate rating using the formula PD = 1 – PI. 

The polymorphism information content (PIC) (16) and deviance from Hardy–Weinberg equilibrium (HWE) (17) were computed with Cervus 3.0.7 (18).

## 3. Results

STR markers were chosen for the *KCNQ1 *gene using the TRF and SERV programs. All of the reported STR markers in the map viewer were dinucleotide repeats. To reduce the chance of stutter bands being formed during the PCR, the markers were selected across tetra- or pentanucleotide tandem repeats that connect to the *KCNQ1* region of chromosome 11 (19). To reduce the probability of a meiosis recombination incident, the markers were selected using a distance of less than 1.4 Mb upstream (D11SU0.6, D11SU2.2, D11SU10.9) and downstream (D11SD13.6, D11SD8.3) of the gene; thus, an intragenic marker (D11SI) was selected.

The Iranian population’s allele frequencies and genetic analysis data (statistical characteristics) for STR markers are shown in Table 3 and Table 4, respectively.

**Table 3:** Distribution of observed allele frequencies of 6 STR loci located in the flanking regions of the KCNQ1 gene (n = 60).

Allele number	D11SU10.9		D11SU2.2		D11SU0.6		D11SI		D11SD8.3		D11SD13.6		Allele size	Frequency	Allele size	Frequency	Allele size	Frequency	Allele size	Frequency	Allele size	Frequency	Allele size	Frequency
	1	326	0.058	295	0.017	165	0.025	82	0.117	279	0.025	120	0.008
2	330	0.033	302	0.008	173	0.175	85	0.042	284	0.225	206	0.017
3	331	0.067	303	0.008	176	0.008	86	0.025	287	0.017	216	0.300
4	332	0.008	304	0.050	177	0.692	101	0.017	289	0.250	219	0.142
5	333	0.017	305	0.008	181	0.067	102	0.008	292	0.117	220	0.208
6	335	0.517	306	0.008	185	0.025	105	0.008	294	0.100	221	0.008
7	336	0.017	308	0.025	189	0.008	107	0.008	297	0.042	223	0.083
8	339	0.250	310	0.008			111	0.200	299	0.008	224	0.092
9	342	0.017	311	0.017			115	0.400	302	0.100	227	0.100
10	343	0.008	312	0.075			119	0.150	306	0.050	231	0.008
11	352	0.008	314	0.200			123	0.017	307	0.017	247	0.017
12			315	0.375			215	0.008	308	0.008	316	0.008
13			317	0.008					311	0.008	320	0.008
14			319	0.083					312	0.008		
15			321	0.017					316	0.017		
16			323	0.042					320	0.008		
17			342	0.008								
18			345	0.042								

**Table 4:** Statistical characteristics of 6 STR loci located in the flanking regions of the KCNQ1 gene (n = 60).

	Na	He	Ho	PI	PD	PIC	PE	HWEexpected P-value
D11SU10.9	11	0.661	0.60	0.15	0.85	0.620	0.635	0.014
D11SU2.2	18	0.799	0.60	0.06	0.94	0.780	0.830	0.20
D11SU0.6	7	0.485	0.533	0.30	0.70	0.450	0.450	0.88
D11SI	12	0.761	0.70	0.09	0.95	0.731	0.757	0.55
D11SD8.3	16	0.847	0.48	0.04	0.96	0.831	0.872	<0.0002
D11SD13.6	13	0.820	0.617	0.05	0.95	0.799	0.832	0.04

He: expected heterozygosity, Ho: observed heterozygosity, PI: probability of identity, PD: power of discrimination, PIC: polymorphic information content, PE: power of exclusion, HWE: Hardy–Weinberg equilibrium.

## 4. Discussion

In total, 77 alleles were detected and the average number of alleles per locus was 12.8. For the D11SU10.9, D11SU2.2, D11SU0.6, D11SI, D11SD8.3, and D11SD13.6 STR markers, fragment assay showed 11, 18, 7, 12, 16 and 13 alleles, respectively. The D11SU2.2 locus with a frequency of 18 alleles was the most polymorphic STR marker. The observed heterozygosity (Ho) was between 0.48 at D11SD8.3 and 0.70 at the D11SI marker. The most frequently detected alleles were 177, 115, and 315 bp for the D11SU0.6, D11SI, and D11SU2.2 markers. The highest PI was 0.30 at D11SU0.6, and the lowest was 0.04 at the D11SD8.3 locus. GenALEx 6.502 showed that the PE was 0.872 and 0.450 at the D11SD8.3 and D11SU0.6 loci, respectively. The power of discrimination was 0.70 at D11SU0.6 and more than 0.92 at all of the other loci. Although 5 out of 6 loci had a PIC above 0.731, the D11SD8.3 marker with content of 0.831 was the most informative locus. According to Cervus 3.0, except for the D11SD8.3 locus, all loci were relevant to HWE, with a probability of less than 0.0002. This revealed that none of the loci except for D11SD8.3 had significant deviations from HWE. This may be due to either probable genotyping and laboratory slips or population stratum (20). 

Our study’s conclusion revealed the novel STRs that show a high rate of informativity and high degree of variability that make STR markers very efficient for haplotype analysis and human identity testing. This method is easy to use for detecting the multiplex reproduction pattern, and also a cost-effective method for detection of disease-causative genes and the prenatal diagnosis of heterogeneous cardiovascular diseases compared to direct sequencing, which is usually time consuming and too expensive.

## Acknowledgment

This research was supported by the Pasteur Institute of Iran (Grant Number 824 partially and PhD grant from Education Office, Pasteur Institute of Iran).
